# In vitro determination of genotoxicity and cytotoxicity induced by stainless steel brackets with and without surface coating in cultures of oral mucosal cells

**DOI:** 10.1186/s12903-024-04976-2

**Published:** 2024-10-16

**Authors:** Dhruv Ahuja, Nidhin Philip Jose, Rozy Kamal, Vinaya Panduranga, Supriya Nambiar, Arun M. Isloor

**Affiliations:** 1 Department of Orthodontics and Dentofacial Orthopedics, Manav Rachna Dental College, Faridabad Manav Rachna International Institute of Research and Studies(MRIIRS), Faridabad, Haryana 121004 India; 2 Department of Orthodontics and Dentofacial Orthopedics, Manipal College of Dental Sciences Mangalore, Manipal Academy of Higher Education, Manipal, Karnataka 576104 India; 3 Department of Nuclear Medicine, Manipal College of Health Professions, Manipal Manipal Academy of Higher Education, Manipal, Karnataka India; 4 Department of Chemistry, National Institute of Technology Karnataka, Surathkal, Mangalore, India

**Keywords:** Orthodontic brackets, Cytotoxicity, Genotoxicity, Corrosion, Oral mucosal cell, Biocompatibility

## Abstract

**Background:**

Orthodontics is a speciality of dentistry that uses a plethora of devices made from myriad materials to manage various malocclusions. Prolonged contact of orthodontic appliances with oral tissues can lead to cellular damage, highlighting the need for biocompatible materials to mitigate health risks.

**Objectives:**

To analyze the genotoxicity and cytotoxicity produced by metal brackets and coated metallic brackets with polymeric and nanoparticle coatings in oral mucosal cells.

**Materials & methods:**

The current study compares the toxicity of 3 different types of orthodontic brackets with control groups of oral mucosal cells. Each of the three treatment groups consisted of 10 samples of orthodontic brackets: stainless steel brackets(Group 1), nanoparticle-coated brackets(Group 2), and polymeric-coated brackets(Group 3) exposed to corrosion eluates employing an oral biomimicry model. Two types of oral mucosal cells- Human Gingival Fibroblasts and Buccal Epithelial Cells were used to study the cytotoxic and/or genotoxic effects of the elutes. Intergroup comparisons were conducted using one-way analysis of variance, while scanning electron microscopy evaluated surface characteristic.

**Results:**

The interaction between metal ions and oral mucosal cells showed no statistically significant difference for toxicity assays between the three groups(*p* > 0.005). However, polymeric and nanoparticle-coated groups showed reduced cellular differentiation when compared with conventional stainless-steel brackets.

**Conclusion:**

This in-vitro study shows that polymeric or nanoparticle coating of conventional metal brackets aids in enhancing corrosion-resistant characteristics of orthodontic appliances and reduces the toxic oral environment created by metal release in the oral cavity.

## Introduction

Orthodontic treatment with intraoral fixed appliance therapy includes bands, brackets, arch wires, and auxiliaries which are formed by various types of metals. These metal alloys consist of nickel-titanium, stainless steel, chromium, or nickel-cobalt alloys which are manufactured with high corrosion resistance [1]. Each orthodontic technique includes brackets that are built with adequate designs and different materials with various alloys and polymer combinations for manufacturing. The stainless-steel alloys commonly used consist of 5–10% nickel (Ni), 15–25% chromium (Cr), and around 40 to 60% cobalt (Co) and iron(Fe) [2, 3]. Usage of orthodontic fixed appliances causes metallic ions to release like chromium, cobalt, iron and nickel into the oral cavity, this metal release with orthodontic therapy is of clinical concern. As these metal alloys come in contact with oral tissues they should not induce cytotoxic, allergic, inflammatory, or genotoxic reactions. Though the combination of metal alloys used during treatment is biocompatible these alloys release ions that are of significant clinical concern as the metals are reacted with electrolytes in the oral cavity which includes saliva, acidic beverages, food items, etc. These produce electrochemical reactions with oral tissues causing biodegradation and corrosion, hence altering the biocompatibility of the metal alloys [4]. 

Due to corrosion, metal compounds can indirectly or directly affect oral tissues and can alter the structures within cells at the DNA level by generating carcinogenic or mutagenic effects [4]. These results are because of the collection, absorption & entry of free radicals intra-orally and other body tissue (intestinal, gingival, respiratory, and cutaneous system epithelial tissue) [5]. A few metallic alloy components, especially nickel, chromium & cobalt have adverse effects and can cause disturbances, including immunological sensibility, hypersensibility, dermatitis, gingivitis, hyperplasia, and can cause asthma. Elemental Cobalt is capable of causing mitosis, Nickel can cause inhibition of cellular proliferation [6]. Both of these metals are considered carcinogenic because they are capable of inducing detrimental effects in oral mucosal cells like human gingival fibroblast cells and human buccal epithelial cells while affecting their morphology and proliferation [7–9]. 

Research and literature in this field include many in-vivo [10–13] and in-vitro [14–17] studies documenting the consequence of corrosion on orthodontic material along with the indisputable leaching of metal ions. Research studies have reported that the ageing of a metallic alloy holds an elemental knowledge of its biocompatibility, as the leaching of ions from the alloy almost always causes adverse biological effects which include allergy, mutagenicity, toxicity, and carcinogenic effects [18]. 

The contemporary knowledge of the molecular and cellular mechanisms of toxicity associated with metals may lead to some concerns involving appliances in orthodontic dentistry [19, 20]. Due to prolonged contact of metal with the oral tissues and its corrosive characteristics results in the leaching of different types and quantities of metal ions [21]. Most of the research on metal ions leached from orthodontic metallic compounds show that they are much below the recommended amount of daily dietary intakes of chromium and nickel and this could be a false assurance of safety [20], as low level of chromium ions alters cellular morphology and metabolism and also produces DNA instability [22, 23]. Moreover, some studies have reported biological toxicity in patients undergoing orthodontic treatment [24, 25]. However, the controversy of metal ions released with orthodontic material in oral mucosa continues.

To date, Titanium is the most biocompatible material, used in the manufacturing of materials in dentistry and fixed orthodontic therapy, it has excellent mechanical properties, good biocompatibility, and high corrosion resistance [26]. On the other hand, Polydopamine (PDA) is a new polymer with interesting characteristics including free-radical scavenging, antioxidant activity, strong metal-ion chelation, and high photothermal conversion efficiency [8]. Both of these compounds show antimicrobial and corrosion resistance properties, also these form coordination bonds when coated with different metal ions, hence altering and enhancing the properties of different metal alloys.

It is essential to manifest a quick and in-vitro assessment to evaluate the biocompatible biomaterials used clinically in orthodontic dentistry. Hence, this study analyses and compares cytotoxicity and genotoxicity which are induced by metal corrosion from various coated and non-coated brackets in human gingival fibroblasts and human buccal epithelial cells. The null hypothesis, in the context of this in-vitro study, is to assess whether the metal ion release with an orthodontic appliance does not induce toxicity. As the oral environment during orthodontic treatment plays a pivotal role in causing degradation and corrosion due to changes in properties of metal alloys this warrants special consideration regarding the biocompatibility of different metals used for orthodontic treatment, due to unbefitting evidence, the objective of this study is to assess corrosion of metals acquired from orthodontic brackets available commercially produces genetic or cytoplasmic damage in oral cavity to predict the risks associated with the process of corrosion occurring during fixed orthodontic therapy.

## Materials and methods

This is an interdisciplinary case-control prospective study, initiated following the acquisition of ethical approval (Ref No: 20088). The study utilized a total of 45 orthodontic bracket samples, with 15 samples in each group, wherein toxicity of 3 different types of orthodontic brackets- Stainless steel brackets (*Group 1*), Titanium Dioxide coated stainless steel brackets(*Group 2*), and Stainless steel brackets with Polydopamine coating (*Group 3*) was compared with control groups of oral mucosal cells.

### Sample size calculation

**Fig. 1:**
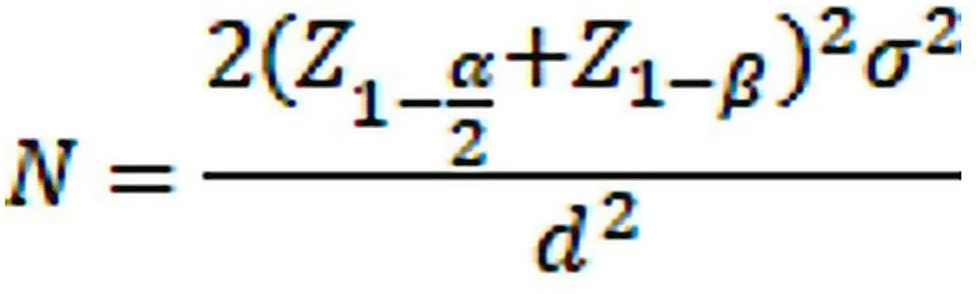
Sample size formula

The sample size was calculated as *n* = 45 (*n* = 5 per group/cell line) for comparing three groups with two different type of cell lines and *n* = 15 for surface characteristic evaluation for three groups.(Fig. 1) The analysis aimed to determine a clinically relevant difference (d) of 0.03 with a power of 90% and an alpha error rate of 1%. This sample size calculation considered a standard deviation (SD) of 0.01 and applied to two different cell types being compared and further experiments were conducted with each sample run in triplicates [4]. 

### Sample preparation

The commercially available stainless-steel orthodontic brackets(Forestadent- Orthosystems, India) The study involved autoclaving brackets with a slot size of 0.022” x 0.028”, a width of 3 mm, and an 80-gauge mesh. Out of the total, 15 brackets were utilized for examining surface characteristics, while the remaining 30 were employed in toxicity assays. Uncoated brackets were used as a control group. For the coated orthodontic brackets, a chemical coating procedure was used. This procedure involved placing 20 mL of Milli-Q water in a Kjeldahl flask. The pH of the water was adjusted to 8.8 using tris base. Two beakers were then taken, and 20 mL of Milli-Q water was added to each beaker. Finally, 4 milligrams of polydopamine was added to each beaker, which served as a binder [27]. In the first beaker, 5 mg of TiO2 granular nanoscale particles were added to the solution in a 1:0.5 ratio. The second beaker contained only 4 mg of polydopamine along with the brackets. A magnetic stir bar was placed in each beaker, and the mixtures were stirred at 300 rpm for 8–12 h at room temperature. Afterward, the samples were retrieved from the solution and placed in a vacuum chamber. They were then transferred to labelled plastic covers for storage until needed [28, 29]. 

### Evaluation of surface characteristics

Following the coating of the orthodontic brackets, their surface topography was assessed using scanning electron microscopy (SEM). The SEM analysis, conducted with an EVO MA18 equipped with Oxford EDS (X-act), examined the microstructure of the coated brackets at a voltage of 10 kV and a magnification of 2500x. Following the coating of the orthodontic brackets, their surface topography was assessed using scanning electron microscopy (SEM) and surface roughness was measured with atomic force microscopy (AFM). The SEM analysis, conducted with an EVO MA18 equipped with Oxford EDS (X-act), examined the microstructure of the coated brackets at a voltage of 10 kV and a magnification of 2500x. For surface roughness evaluation, five samples from each of the 45 orthodontic brackets were analyzed quantitatively using a Flex-Axiom AFM from Nanosurf, Switzerland. The root mean square (RMS) roughness was calculated and averaged, with images revealing the surface roughness over an area of 101 μm² [30]. 

### Preparation of cell cultures

Cell cultures of Human Gingival Fibroblasts(HGF-1) and Buccal Epithelial Cells(hTERT-OME) were derived from cell lines of gingival and epithelial cells. Primary cell cultures were grown in 25-cm2 culture flasks(Himedia) to confluence using Dulbecco’s Modified Eagle Medium (DMEM) for gingival fibroblasts and Modified Eagle Medium (MEM) for epithelial cells, which were further supplemented with 10% fetal bovine serum (FBS) and antibiotics antimycotic solution. Cell viability was analysed after collection, through cell suspension count for two types of cells which was noted using a Neubauer chamber.

### Preparation of corrosion eluates

The eluates prepared for the corrosion process consisted of sodium chloride (0.1 M) and acetic acid (0.1 M) solution(Sigma-Aldrich, Merk, India). Calculated amounts of NaCl and CH_3_COOH were mixed in distilled water and stirred with magnetic beads at 350 rpm at room temperature for 10 min. Each bracket was submitted to the 2 ml of corrosion solution at room temperature for 35 days and further sterilized with ultraviolet light before use [4, 9]. 

### Exposure of cell cultures to corrosion eluates

**Fig. 2:**
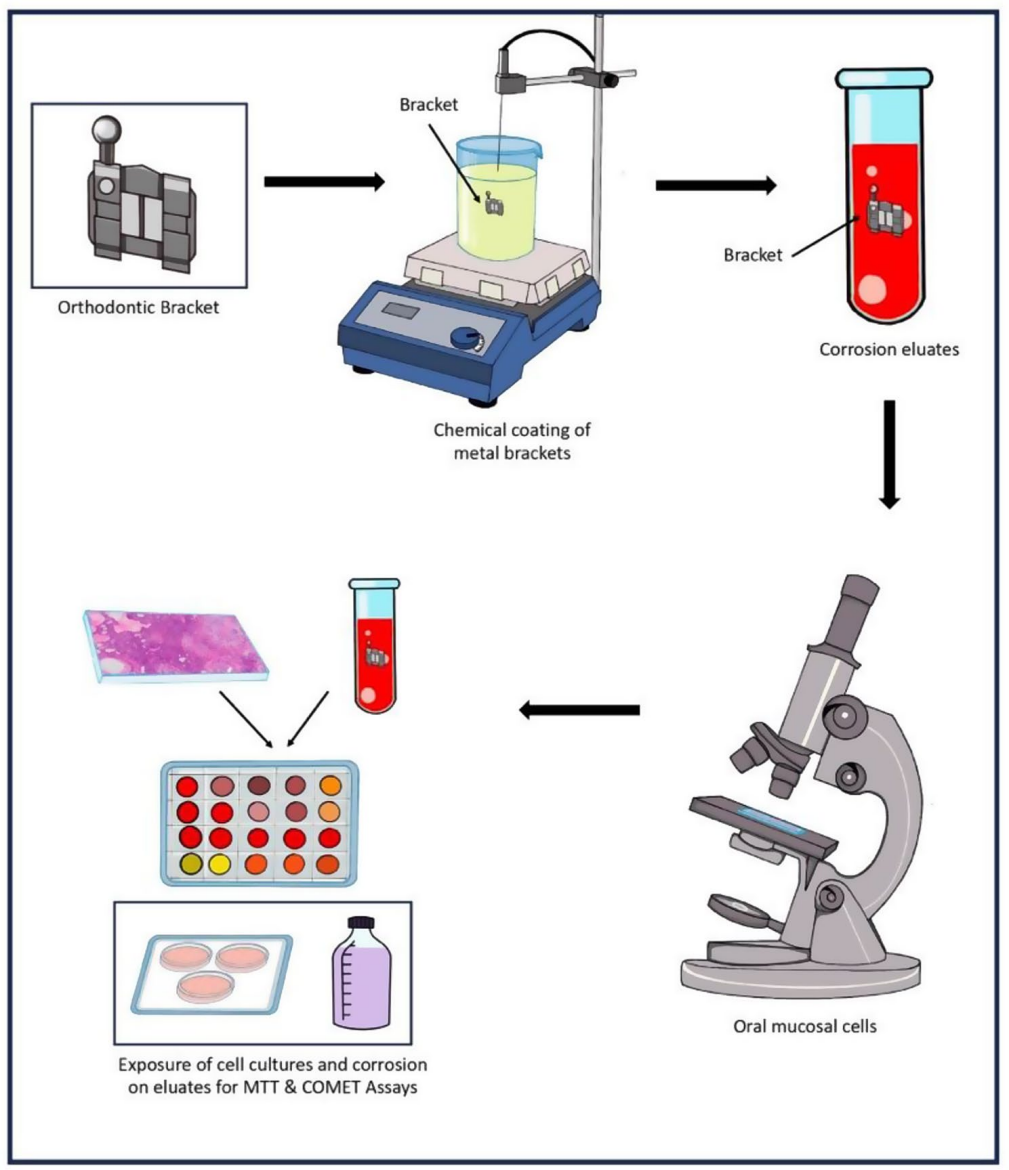
Sample preparation and cell culture for MTT and Comet Assay

The obtained corrosion eluate was exposed to the two types of cells for cytotoxicity(colorimetric assay), measuring the reduction of 3-(4, 5-dimethylthiasol-2-yl)-2,4,-diphenyltetrazolium bromide(MTT) by mitochondrial succinate dehydrogenase and genotoxicity experiments using single cell gel electrophoresis (comet) assay, the alkaline version, to evaluate the magnitude of DNA damage. In addition, positive control, cells treated with acetic acid/ sodium chloride solution without brackets and negative control, cells treated with saline were used in the experiments. A volume of 10 µL of cells (around 10,000 cells) was individually added to each final eluate solution and incubated for 30 min at 37 °C. Following this exposure, the cells were rinsed with phosphate-buffered saline (PBS). The negative control was exposed to the same solution used for the corrosion process, also for 30 min at 37 °C. (Fig. 2)

### 3-(4,5-Dimethylthiazol-2-yl)-2,5-diphenyltetrazolium bromide assay (MTT Assay)

The experiment was carried out in triplicate. Corrosion eluate was diluted 100 times and used for different assays. Cultured cells were removed from flasks and 10µL of 100 times diluted equates was added to each well in a 96well tissue culture at density (1 × 103/well). After cells had been seeded and allowed to adhere overnight, 10 µL of the 100-fold diluted corrosion eluate was added to each well. A corrosion solution without brackets was used as a negative control. 96-well cell culture plates were used to carry out all the chemical exposures. The cells were incubated with the corrosion eluate for a period of 24 h. During this time, the cells were exposed to the eluate, and any potential cytotoxic effects were manifested. After the incubation period, MTT solution 5 mg/mL in PBS or culture medium were added to each well. The plate was then incubated again for 2–4 h. During this time, viable cells metabolized the MTT and further solubilization or dissolution of purple formazan crystals were checked by the metabolic activity of viable cells and measured by spectrophotometrical absorbance of the samples using a microplate reader (ELISA).

### Single cell gel electrophoresis assay (Comet assay)

Counted cells were re-suspended(10^3^ cells/ well) in the growth medium with 10% fetal bovine serum (FBS). Cell suspension aliquots of 10 µL of the sample were added in each well of 96-well plates and then incubated in a humidified 5% CO2 incubator at 37 °C for 24 h. Cultured cells were centrifuged, after incubation, and then the Giemsa staining protocol was followed to visualize cells under a bright-field microscope. Replicated three slides of different groups were evaluated using visual scoring at 20x and 40x magnification under a contrast phase microscope for DNA changes.

### DNA laddering assay

DNA ladder assay was carried out after cell lines were cultured with 10% FBS and antibiotics in a humidified incubator with an atmosphere of 95% air and 5% CO2 at 37 °C. Induction of apoptosis occurred by 500 µM H2O2 for 48 h. DNA ladder assay was performed as a confirmatory finding for the apoptosis of cells. The image-based data obtained from the experiments were expressed to determine the DNA cleavage, programmed cell death, or apoptosis detection.

### Statistical analysis

The data were organized and tabulated using Microsoft Excel. Statistical analyses were performed using IBM SPSS Statistics for Windows, Version 25.0. Armonk, NY; IBM Corp. The results were expressed in means ± standard deviation. All the experiments were expressed in triplicates. The intergroup comparison was done using the one-way analysis of variance (ANOVA) followed by the Posthoc Tukey test with p-values < 0.05 were considered statistically significant. Scanning electron microscopy was used to verify the presence of the coatings.

### Cytotoxicity assay data

Agilent Gen5 3.12 computer software program with optimal density readings at 550 nm was used to calculate cell viability percentages and the results were represented as means ± standard deviation. The cell viability percentages were represented graphically.

### Genotoxicity assay data

In the assessment of comet assay, comets were randomly selected from replicated slides using image analysis(image focus alpha) for DNA migration. DNA fragmentation was noted through DNA ladder assay as a confirmatory finding for apoptosis of cells. The image-based data obtained from the experiments were expressed to determine the DNA cleavage, programmed cell death, or apoptosis detection.

## Results

### Surface characteristics

**Fig. 3:**
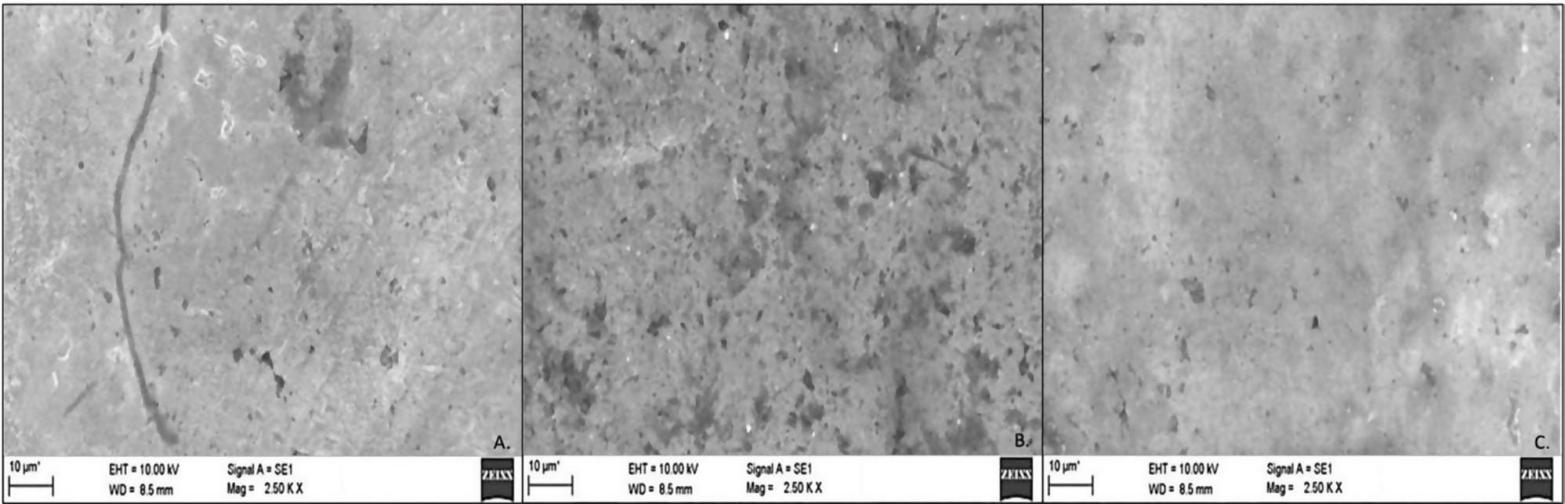
SEM images: Uncoated brackets (**A**.), Titanium dioxide coated brackets (**B**.), Polydopamine coated brackets (**C**.)

The SEM analysis confirmed the elemental composition of the coating, verifying the presence and distribution of the nanoparticles across the bracket surface. (Fig. 3)

### 3-(4,5-Dimethylthiazol-2-yl)-2,5-diphenyltetrazolium bromide assay /MTT assay

The results of the cytotoxicity are presented, where the control group had maximum cell viability% as there were no brackets. In the treatment group, a comparison of the parameter Gingival fibroblasts (cell viability%) between the three groups showed no statistically significant difference between the groups (test value of 0.454 and p-value of 0.643). The highest mean values were seen in Titanium dioxide (76.17 ± 17.3) followed by Polydopamine (74.46 ± 21.76) and Stainless steel (70.68 ± 8.53). Subgroup analysis using the posthoc Tukey HSD test shows that the largest difference between the groups was noted between SS vs. TiO2 (5.487) which was not significant, followed by SS vs. PDA (3.783) and PDA vs. TiO2 (1.704).

Similar findings were seen with Buccal Epithelial Cells where cell viability% values Comparison of the parameter Buccal Epithelial cells (cell viability%), showed no statistically significant difference between the groups (test value of 0.29and p-value of 0.751). The highest mean values were seen in TiO2 (56.66 ± 17.23) followed by PDA (53.17 ± 18.18) and SS (51.43 ± 10.27). Subgroup analysis using posthoc Tukey HSD test shows that the largest difference between the groups was noted between SS vs. TiO2 (5.226) which was not significant, followed by PDA vs. TiO2 (3.486, not significant) and SS vs. PDA (1.74, not significant). (Table 1)

**Table 1:** Cell viability% of human gingival fibroblasts and buccal epithelial cells with different groups

	SS (*N* = 10) Mean ± SD	PDA (*N* = 10) Mean ± SD	TiO2 (*N* = 10) Mean ± SD	F / Welch Statistics (* represents welch test)	*P* value	SS vs. PDA Difference (*p* value)	SS vs. TiO2 Difference (*p* value)	PDA vs. TiO2 Difference (*p* value)
**Gingival fibroblasts(cell viability%)**	70.68 ± 8.53	74.46 ± 21.76	76.17 ± 17.3	0.454 *	0.643	-3.78(0.87)	-5.49(0.748)	-1.7(0.972)
**Buccal Epithelial cells(cell viability%)**	51.43 ± 10.27	53.17 ± 18.18	56.66 ± 17.23	0.29	0.751	-1.74(0.966)	-5.23(0.738)	-3.49(0.873)

**Graph 1:**
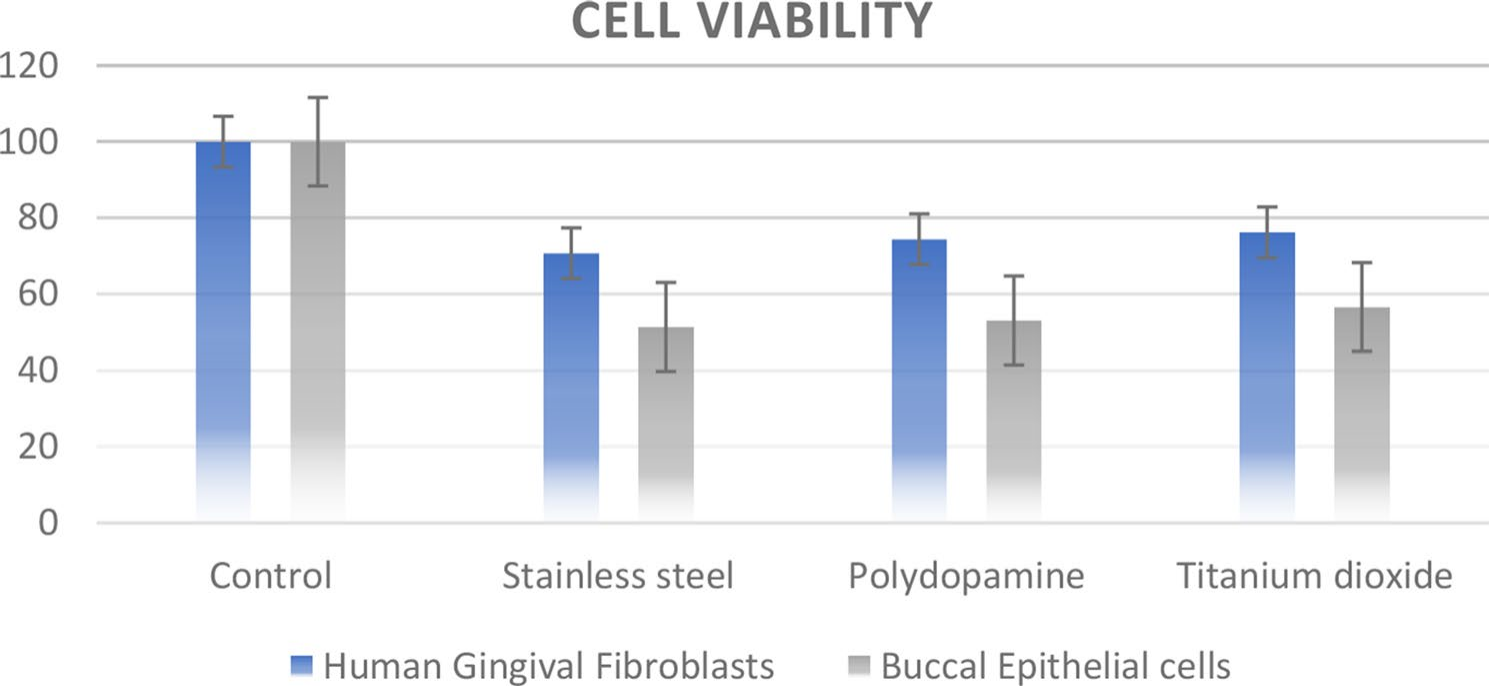
Cell viability% of Human Gingival Fibroblasts and Buccal Epithelial cells in different groups

However, a noteworthy reduction of viable cells was recorded in all the groups when compared to the control. Polydopamine and Stainless steel showed almost similar cell viability indicating no significant reduction in toxicity with the stainless steel group showing a reduced cell viability percentage than other groups. The titanium dioxide group when compared, showed more cell viability indicating reduced cellular metabolic activity. (Graph 1)

### Single cell gel electrophoresis assay /comet assay

Comet assay images represent control and treated Human Gingival Fibroblasts and Buccal Epithelial Cells making use of the Giemsa staining protocol. Results suggested distribution of cells by comet category was based on the metal composition and the leached metal ions due to corrosion and embedded in the cellular matrix of cells. In the control, the majority of cells were found to be intact and the titanium dioxide group showed few cells with cellular alteration, whereas Stainless steel and Polydopamine groups showed nuclear diffusion and cellular damage. However, the image-based data obtained was not significant but the Stainless steel and Polydopamine group showed increased nuclear damage of cells, and the least amount of damaged cell percentage was seen in the Titanium dioxide group.

**Fig. 4:**
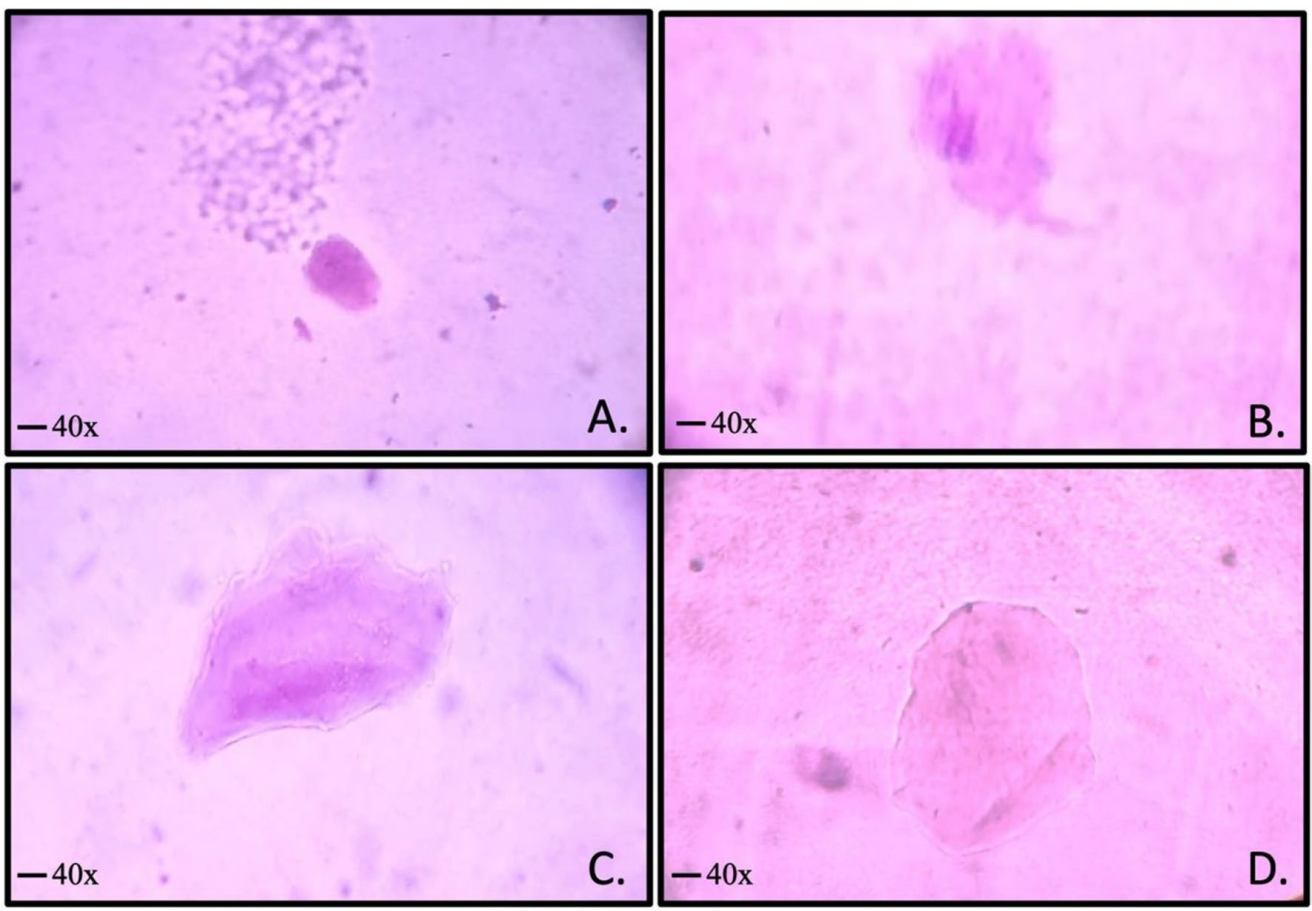
Representative genotoxic assay images from control (**A**), Stainless steel (**B**), Polydopamine (**C**) and Titanium dioxide (**D**)

These findings were similar for both types of cells where there was no significant amount of comet tails or damaged DNA of cells. Apoptosis and diffusion of nuclear material of Buccal Epithelial cells were evident in the stainless steel group followed by the polydopamine group and least for the Titanium dioxide group. (Fig. 4)

**Fig. 5:**
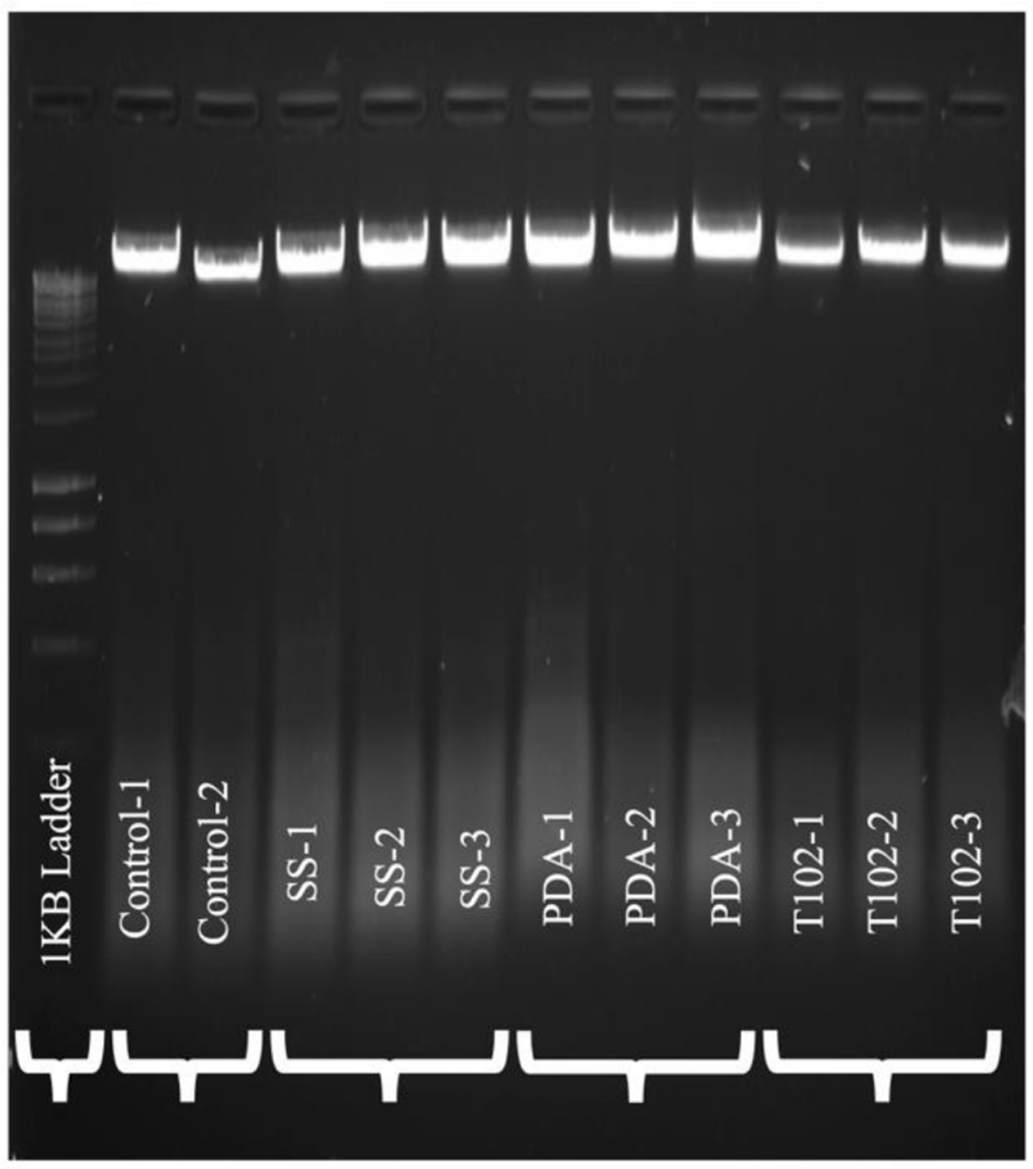
DNA fragmentation forming a ladder in different groups corresponding to oral mucosal cells

Delineated images of the comet assay for control and Buccal Epithelial Cells showed no significant damage to DNA in cells when compared among the groups. However, the groups showed increased diffusion of the nucleus compared to the control cells. The generated data from these investigations demonstrated a gradual variation and diffusion of nuclear material. Also, the findings of Human Gingival Fibroblast assessed by DNA fragmentation assay indicate infinitesimal apoptosis of cells with metal ions released in corrosion solution with no significant differences between the groups. (Fig. 5)

## Discussion

This study aims to assess the cytotoxic and genotoxic damage in oral mucosal cells produced by corrosion eluates which were acquired from fixed orthodontic appliances using Human Gingival Fibroblasts and Buccal Epithelial Cells in vitro. This is so far the novel approach to test different types of polymeric and nanoparticle coating on orthodontic appliances to assess the biocompatibility of the metals that are present in the oral cavity for an individual undergoing orthodontic therapy for a year or more.

The results demonstrated from the two toxicity assays are more or less similar to the effects noticed in vivo. Human Gingival Fibroblasts are commonly tested cells for studies whereas Buccal Epithelial Cells have enduring contact with orthodontic appliances throughout the treatment, these cells were used in this study because their cellular types are easily noticeable and characterized concerning their growth pattern [31]. Also these are the cell types that have potential risk of getting affected by carcinogenic metals.

Based on cytotoxic data obtained the study showed indistinguishable differences between the viability of cells among the groups though the data obtained showed increased cell death and toxicity with stainless steel as these contain chromium and nickel which are known potential carcinogenic agents, the polymeric-coated metals showed a slightly reduced amount of cell metabolic activity than stainless steel when compared with control group, this is also noted in other studies as polydopamine is known to have biocompatible properties. On the other hand, Titanium coated group has shown the least cytoplasmic damage than other groups which is correlated to its original biocompatible properties [32]. 

The obtained genotoxic data from the study indicated that single-cell gel electrophoresis assay in exploratory conditions inconsequentially detected the existence of nuclear diffusion of a few cells after treatment with eluates formed from corrosion of commercially available brackets coated with a polymer and a nanoparticle at all experimental periods established. The genotoxic experiments showed an insignificant amount of cellular alterations associated with both types of cells among the groups.

The research studies conducted in this field have denoted that corrosion eluates procured from materials and implants used in dentistry were not considered genotoxic, and titanium being the most biocompatible nanoparticle does not exert DNA injury in vivo [33, 34]. Many studies have postulated that patients undergoing orthodontic therapy do not show primary damage to DNA or genetic material in oral mucosa cells [33]. However, few studies have reported cytotoxic and genotoxic damage produced by orthodontic appliances over a period of time in Human Gingival Fibroblasts and Buccal Epithelial Cells as evaluated by colorimetric assay and single gel electrophoresis assay in vivo [2]. In the past few years, studies have been done and standardized the genotoxic assay in human epithelial cells like gastric cells and urothelial cells [35, 36] Like other epithelial cells, oral cells including gingival and buccal epithelium consist of stratified squamous epithelium which in particular undergoes differentiation terminally to formulate a barrier which protects the cells. This barrier is a small proline-rich protein called cell envelope, which provides a barrier in the oral cavity against a very hostile oral environment, this barrier provides defiance of oral cells to lysis [37]. In such a manner, cell lysis using a proteinase K or trypsin-assisted procedure is needed for assays to be performed for cellular, metabolic, or genetic damage.

The exploratory studies have shown results using a genotoxic assay in cells of animals denoted that few oral mucosal cells yielded cellular DNA damage as oral cells sustained massive damage to DNA or nuclear material with subsequent disintegration. Hence, the comet assay, which is performed commonly, may not provide genotoxicity monitoring in oral mucosa cells because of its cell characteristics until or unless a significant carcinogenic agent is involved [38]. The existence of any metals within the oral cavity environment undergoes corrosion due to organometallic compound formation, which is dependent on metal and its alloy composition, pH, temperature, and humidity in the oral cavity environment [39, 40]. 

In vivo [41] and in vitro [42] studies have assessed the metal release like chromium, nickel, and iron in orthodontic dentistry. Studies have shown that metal ion leaching transpires during the initial phase of fixed orthodontic therapy and produces cytogenic, mutagenic, and immunogenic effects. In particular, Nickel- Titanium induces cell death or apoptosis by inhibition of Ni^2+^ and involves caspase-3 activation among Human Gingival Fibroblasts and Buccal Epithelial Cells [43]. The most cytotoxic element is Nickel used in orthodontic appliances which is followed by chromium, as these ions penetrate cells and affect the function and morphology leading to hypersensitivity reactions in the oral cavity [44]. Stainless steel brackets also contain Manganese which is a corrosion product that causes mitochondrial dysfunction and results in cellular oxidative stress producing glycosaminoglycans (GAG) accumulation intra and extracellularly affecting cellular differentiation, adhesion, migration, and growth [45]. 

Considering the various adverse effects of stainless steel and alloys with pure titanium which are more stable and inert to the environment have been introduced. With advancements in technology newer polymeric-based materials have been introduced such as Polydopamine, both these materials have better properties to overcome the side effects of stainless steel. Titanium and Polydopamine have shown improved biocompatibility, and high characteristics of corrosion resistance, and do not bring about any inflammatory responses [46, 47]. 

However, newer nanoparticle-coated brackets have been reported to show increased biocompatible properties in human cells due to altered metal alloy composition, these coated brackets eliminate impurities or contamination associated with the manufacturing process. Recently studies have shown the biocompatibility of polydopamine which is an inert polymer, hence we have used polydopamine coating for assessing the effectiveness of polymer with conventional brackets, our study showed no significance between the groups however it has yielded that polymeric surface coating is effective and more biocompatible in the oral environment.

According to the available literature so far, this study approach has not been addressed where two different types of coating on the conventional metallic orthodontic brackets are compared, the polymeric and nanoparticle coatings in cultures of two types of oral mucosal cells, this in-vitro study has provided clinically significant information and clarifies the mechanisms of toxicity associated with metal components in oral environment.

### Limitation(s)

The primary limitation of this study is it’s in vitro nature, which may not fully capture the complexity of in vivo biological interactions and responses, thereby necessitating further research.

## Conclusion

Oral mucosal cells showed no significant difference in cell viability between the coated and uncoated bracket groups, though the stainless steel brackets have shown increased cellular alteration followed by polymeric-coated brackets and nanoparticle-coated brackets. The fate of changes in the oral cavity due to orthodontic appliance needs follow-up, as there are continuous biological changes taking place in the oral environment during orthodontic therapy. This in-vitro study model is helpful for further research of cellular and metabolic damage in oral mucosal cells induced by metals or biomaterials used in orthodontic therapy before their introduction into orthodontic dentistry.

## Data Availability

data is provided within the manuscript or supplementary information files.

## Declarations

### Generative AI in scientific writing

During the preparation of this work, the author(s) have not used any AI tool. The author(s) reviewed and edited the content as needed and take(s) full responsibility for the content of the publication.
